# Supplementary material for: Extended Spectrum Beta-Lactamase-Resistant Determinants among Carbapenem-Resistant Enterobacteriaceae from Beef Cattle in the North West Province, South Africa: A Critical Assessment of Their Possible Public Health Implications
Source: Antibiotics (Basel). 2020 Nov 17;9(11):820. doi: 10.3390/antibiotics9110820 (PMC7698526; doi:10.3390/antibiotics9110820)
Supplement: Supplementary file 1 [file antibiotics-09-00820-s001.pdf]

antibiotics

Article

Extended Spectrum Beta-Lactamase-Resistant Determinants among Carbapenem-Resistant *Enterobacteriaceae* from Beef Cattle in the North West Province, South Africa: A Critical Assessment of Their Possible Public Health Implications

Lungisile Tshitshi [1,2], Madira Coutlyne Manganyi [3], Peter Kotsoana Montso [4], Moses Mbewe [2] and Collins Njie Ateba [1,4,*]

1. Antimicrobial Resistance and Phage Biocontrol Research Group, Department of Microbiology, School of Biological Sciences, Faculty of Natural and Agricultural Sciences, North West University, Private Bag X2046, Mmabatho 2735, South Africa; Lungisile.Tshitshi@ump.ac.za
2. Faculty of Agriculture and Natural Sciences, University of Mpumalanga, Private Bag X11283, Mbombela 1200, South Africa; Moses.Mbewe@ump.ac.za
3. Unit for Environmental Sciences and Management, North-West University, Potchefstroom 2520, South Africa; madira.manganyi@nwu.ac.za
4. Food Security and Safety Niche Area, Faculty of Natural and Agricultural Sciences, North West University, Private Bag X2046, Mmabatho 2735, South Africa; montsokp@gmail.com

* Correspondence: collins.ateba@nwu.ac.za.

Table S1. Multidrug resistance phenotypes and MAR index of CRE isolates.

Antibiotypes	Multidrug Resistance Phenotypes	Number of Isolates	Number of Antibiotics	MAR Index
AB_1	ATM-CTX-FOX	1	3	0.23
AB_2	A-KF-CAZ	2	3	0.23
AB_3	CPM-EFT-CTX	1	3	0.23
AB_4	ATM-EFT-CAZ	1	3	0.23
AB_5	CPM-ATM-TC	1	3	0.23
AB_6	ATM-A-CIP	1	3	0.23
AB_7	CXM-PRL-CTX	2	3	0.23
AB_8	A-KF-FOX	2	3	0.23
AB_9	PRL-CTX-CAZ	2	3	0.23
AB_10	CXM-ATM-KF-CTX	1	4	0.31
AB_11	EFT-CTX-FOX-AMP	1	4	0.31
AB_12	CXM-ATM-A-CTX	2	4	0.31
AB_13	CXM-PRL-KF-FOX	1	4	0.31
AB_14	ATM-EFT-FOX-AMC	1	4	0.31
AB_15	CPM-PRL-TC-CTX	1	4	0.31
AB_16	EFT-CTX-CAZ-FOX	2	4	0.31
AB_17	CXM-ATM-PRL-CTX	1	4	0.31
AB_18	CXM-ATM-CTX-CAZ	1	4	0.31
AB_19	CPM-ATM-KF-FOX	1	4	0.31
AB_20	ATM-A-TC-CTX	1	4	0.31
AB_21	EFT-A-KF-FOX	1	4	0.31
AB_22	CPM-ATM-EFT-CTX	1	4	0.31

AB_23	CXM-ATM-PRL-CIP	1	4	0.31
AB_24	CXM-ATM-CAZ-CIP	1	4	0.31
AB_25	CXM-ATM-EFT-TC	1	4	0.31
AB_26	ATM-TC-KF-FOX	1	4	0.31
AB_27	A-PRL-KF-FOX	1	4	0.31
AB_28	ATM-CTX-CAZ-CIP	1	4	0.31
AB_29	CXM-ATM-TC-CTX	1	4	0.31
AB_30	ATM-PRL-CTX-FOX	1	4	0.31
AB_31	CXM-CPM-TC-CTX	1	4	0.31
AB_32	PRL-CAZ-FOX-CIP	1	4	0.31
AB_33	CXM-ATM-FOX-CIP	1	4	0.31
AB_34	CXM-ATM-EFT-PRL	1	4	0.31
AB_35	ATM-EFT-PRL-AMC	1	4	0.31
AB_36	EFT-PRL-TC-FOX	1	4	0.31
AB_37	ATM-EFT-A-PRL	1	4	0.31
AB_38	EFT-A-KF-CAZ	1	4	0.31
AB_39	EFT-A-KF-CTX	1	4	0.31
AB_40	EFT-A-CTX-FOX	1	4	0.31
AB_41	EFT-TC-KF-FOX	1	4	0.31
AB_42	ATM-KF-CAZ-FOX	1	4	0.31
AB_43	EFT-CAZ-FOX-AMC	1	4	0.31
AB_44	CXM-KF-CTX-CAZ	1	4	0.31
AB_45	EFT-A-PRL-FOX	1	4	0.31
AB_46	CPM-ATM-EFT-CTX-FOX	1	5	0.38
AB_47	CXM-ATM-EFT-CTX-FOX	1	5	0.38
AB_48	EFT-A-PRL-KF-CTX	1	5	0.38
AB_49	CXM-ATM-PRL-TC-CTX	5	5	0.38
AB_50	CXM-CPM-PRL-CTX-CAZ	1	5	0.38
AB_51	CXM-CPM-ATM-CAZ-FOX	1	5	0.38
AB_52	CXM-PRL-TC-CTX-CAZ	1	5	0.38
AB_53	EFT-PRL-TC-CTX-CAZ	1	5	0.38
AB_54	CXM-PRL-KF-CTX-CAZ	1	5	0.38
AB_55	ATM-EFT-A-TC-CTX	1	5	0.38
AB_56	ATM-TC-KF-CTX-CIP	1	5	0.38
AB_57	CXM-EFT-PRL-TC-CTX	1	5	0.38
AB_58	CXM-CPM-A-PRL-CAZ	1	5	0.38
AB_59	CXM-EFT-A-KF-FOX	1	5	0.38
AB_60	EFT-A-TC-KF-FOX	2	5	0.38
AB_61	CXM-ATM-EFT-KF-FOX	1	5	0.38
AB_62	ATM-TC-KF-CTX-FOX	1	5	0.38
AB_63	CXM-ATM-TC-KF-FOX	1	5	0.38
AB_64	ATM-A-PRL-CAZ-FOX	1	5	0.38
AB_65	CXM-PRL-TC-CAZ-ATM	1	5	0.38
AB_66	EFT-A-PRL-TC-KF	1	5	0.38
AB_67	ATM-EFT-TC-KF-CTX	1	5	0.38
AB_68	CXM-EFT-A-TC-CTX	1	5	0.38
AB_69	EFT-A-PRL-CTX-AMC	1	5	0.38
AB_70	ATM-A-CTX-FOX-CIP	1	5	0.38
AB_71	ATM-TC-CTX-AMC-AMC	1	5	0.38
AB_72	ATM-EFT-PRL-TC-AMC	1	5	0.38

AB_73	CXM-ATM-TC-KF-CTX	1	5	0.38
AB_74	CXM-ATM-A-KF-AMC	1	5	0.38
AB_75	A-PRL-TC-KF-CAZ	1	5	0.38
AB_76	A-PRL-TC-K-CAZ	1	5	0.38
AB_77	CXM-EFT-A-TC-FOX	1	5	0.38
AB_78	A-KF-CTX-CAZ-FOX	1	5	0.38
AB_79	CXM-CPM-EFT-A-CTX-CAZ	1	6	0.46
AB_80	CXM-ATM-EFT-CTX-CAZ-FOX	1	6	0.46
AB_81	CPM-A-PRL-TC-CTX-CAZ	1	6	0.46
AB_82	CPM-A-PRL-CTX-CAZ-FOX	1	6	0.46
AB_83	CXM-EFT-A-PRL-CTX-CAZ	1	6	0.46
AB_84	CXM-EFT-PRL-TC-KF-CAZ	1	6	0.46
AB_85	CXM-CPM-CTX-CAZ-FOX-CIP	1	6	0.46
AB_86	CPM-EFT-A-PRL-CTX-CAZ	1	6	0.46
AB_87	CPM-ATM-PRL-CTX-CAZ-FOX	1	6	0.46
AB_88	CXM-ATM-KF-CTX-CAZ-FOX	1	6	0.46
AB_89	ATM-PRL-CTX-CAZ-CIP-AMC	1	6	0.46
AB_90	ATM-EFT-PRL-KF-FOX-CIP	1	6	0.46
AB_91	CXM-ATM-PRL-CTM-FOX-CIP	1	6	0.46
AB_92	ATM-A-PRL-TC-CTX-CAZ	1	6	0.46
AB_93	ATM-PRL-TC-KF-CTX-CIP	1	6	0.46
AB_94	CXM-CPM-ATM-A-TC-CTX	1	6	0.46
AB_95	CXM-CPM-ATM-A-CAZ-FOX	1	6	0.46
AB_96	CXM-CPM-ATM-PRL-KF-CTX	1	6	0.46
AB_97	CPM-ATM-EFT-KF-CAZ-FOX	1	6	0.46
AB_98	CPM-ATM-KF-CTX-CAZ-FOX	1	6	0.46
AB_99	ATM-TC-KF-CTX-CAZ-FOX	1	6	0.46
AB_100	CXM-A-TC-CTX-CAZ-FOX	1	6	0.46
AB_101	CXM-ATM-A-PRL-TC-FOX	1	6	0.46
AB_102	CXM-ATM-A-PRL-KF-CTX	1	6	0.46
AB_103	CXM-ATM-EFT-TC-CTX-FOX	1	6	0.46
AB_104	EFT-A-PRL-TC-KF-CAZ	1	6	0.46
AB_105	CXM-ATM-EFT-A-KF-CAZ	1	6	0.46
AB_106	EFT-PRL-TC-KF-CTX-CAZ	1	6	0.46
AB_107	ATM-EFT-TC-KF-CTX-CAZ	1	6	0.46
AB_108	CXM-EFT-A-CTX-FOX-CIP	1	6	0.46
AB_109	A-PRL-KF-CTX-CAZ-FOX	1	6	0.46
AB_110	CXM-PRL-TC-KF-CTX-CAZ-AMC	1	7	0.54
AB_111	ATM-EFT-A-PRL-TC-CTX-FOX	1	7	0.54
AB_112	CPM-EFT-A-PRL-KF-CTX-FOX	1	7	0.54

AB_113	CXM-CPM-ATM-A-TC-CTX-CIP	1	7	0.54
AB_114	CXM-CPM-ATM-EFT-PRL-CTX-FOX	1	7	0.54
AB_115	CXM-EFT-A-KF-CTX-CAZ-FOX	2	7	0.54
AB_116	CXM-ATM-EFT-A-PRL-KF-FOX	1	7	0.54
AB_117	CXM-ATM-KF-CTX-CAZ-FOX-CIP	1	7	0.54
AB_118	CXM-ATM-EFT-TC-KF-CAZ-FOX	1	7	0.54
AB_119	CXM-ATM-PRL-TC-KF-CTX-CAZ	1	7	0.54
AB_120	CPM-ATM-PRL-TC-KF-CTX-FOX	1	7	0.54
AB_121	ATM-A-PRL-KF-CTX-FOX-AMC	1	7	0.54
AB_122	CXM-CPM-ATM-PRL-TC-CTX-FOX	1	7	0.54
AB_123	CXM-EFT-A-PRL-KF-CAZ-CIP	1	7	0.54
AB_124	CXM-ATM-EFT-A-CTX-CIP-AMC	1	7	0.54
AB_125	CXM-ATM-EFT-A-TC-KF-CTX	1	7	0.54
AB_126	CXM-EFT-A-TC-KF-CTX-FOX-AMC	1	8	0.62
AB_127	CXM-CPM-ATM-A-PRL-TC-CTX-CAZ	1	8	0.62
AB_128	CXM-CPM-ATM-EFT-A-TC-KF-CTX	1	9	0.69
AB_139	CXM-CPM-ATM-A-PRL-TC-CTX-CAZ-FOX	1	9	0.69

MAR = Multi antibiotic resistance A = Amoxicillin, AMC = Amoxicillin-clavulanate, ATM = Aztreonam, CPM = Cefepime, CTX = Cefotaxime, FOX = Cefoxitin, CAZ = Ceftazidime, CXM = Cefuroxime, EFT = Ceftiofur, KF = Cephalothin, CIP = Ciprofloxacin, PRL = Piperacillin and TC = Ticarcillin; .Average MAR index = 0.40.
